# Imperforate Anus

**Published:** 1874-05-01

**Authors:** E. O. F. Roler

**Affiliations:** Mercy Hospital


### IMPERFORATE ANUS.

THE SUBSTANCE OF A CLINIC BY E. O. F. ROLER, M.D., IN MERCY HOSPITAL, APRIL 14, 1874. REPORTED BY J. KEWLEY.

GENTLEMEN: On the 9th of April, Mrs. —— gave birth to what was, apparently, a perfectly formed and healthy female child. The infant's bowels not having moved, a dose of castor oil was administered a day or so after its birth; catharsis, however, did not follow its administration, but the medicine was after a time ejected from the stomach. An enema was now thrown into the rectum, but was not retained. On the afternoon of the 11th my attention was called to the patient, and on examination I found the rectum to terminate in a cul-de-sac. This kind of malformation is very rare, and in them one of two conditions exists: 1st, The walls of the rectum may be perfectly formed, the cul-de-sac being formed by a constriction of the walls, or, more frequently, by a septum thrown across, bearing the same relation to the rectum as the imperforated hymen does to the vagina. 2d, The rectum may terminate in a pouch before it reaches the anus, being, as it were, too short. In such cases we find a cul-de-sac more or less deep, terminating in the anus; the bottom of the cul-de-sac being separated from the rectum above by perhaps cellular tissue. In the first case, operations have frequently been successful; in the second case, the prognosis is more grave; yet an operation is the last and only hope of saving the life of the child.

This infant, although it has nursed some, is quite emaciated, and has been vomiting biliary matter in considerable quantity; hence little hope can be entertained of its recovery. After each of the students had examined the infant, Prof. Roler wrapped a narrow bandage around a bistoury, with the exception of its sharp point, and then introducing his little finger into the cul-de-sac, as a guide to the instrument, he carefully introduced it, and upon withdrawing the instrument, gases, and a quantity of meconium, escaped.

The infant died, however, during the night of the 14th; and at the autopsy, held on the 15th by D. T. Nelson, M.D., the pathological condition of the rectum was found to correspond with that of the second case enumerated above. The bistoury had pierced the cul-de-sac, and penetrated the bottom of the pouch above. In the ilium was found a small perforation, diagnosed as rupture from over distension of the intestine. The small intestines, with the exception of the duodenum, and a small portion of the jejunum, were highly inflamed and distended. The ascending, and a small portion of the transverse, colon, presented a high state of inflammation; the hepatic flexure having nearly a gangrenous appearance, and in a few days, at the most, would have ruptured. The portion of the liver in contact with the inflamed bowels, was of a deep brown color, almost black; and the gall-bladder was distended with bile. No other pathological lesions or malformations were discovered. Death was undoubtedly caused by the inflammation of the bowels, as a probe could be very easily introduced, at death, into the rectum, through the opening made by the bistoury.

